# Disulfide Engineered Lipase to Enhance the Catalytic Activity: A Structure-Based Approach on BTL2

**DOI:** 10.3390/ijms20215245

**Published:** 2019-10-23

**Authors:** César A. Godoy, Javier Klett, Bruno Di Geronimo, Juan A. Hermoso, José M. Guisán, César Carrasco-López

**Affiliations:** 1 Departamento de Química (LIBB), Grupo de Investigación en Ingeniería de los Procesos Agroalimentarios y Biotecnológicos (GIPAB), Universidad del Valle, C.P. 76001 Cali, Colombia; 2 Experimental Therapeutics Programme, Spanish National Cancer Research Centre (CNIO), C/Melchor Fernández Almagro 3, E-28029 Madrid, Spain; jklett@cnio.es (J.K.); bdigeronimo@ext.cnio.es (B.D.G.); 3 Department of Crystallography and Structural Biology, Institute of Physical Chemistry “Rocasolano” (IQFR-CSIC), E_28006 Madrid, Spain; xjuan@iqfr.csic.es; 4 Departamento de Biocatálisis. Instituto de Catálisis. CSIC. Campus UAM. Cantoblanco. C.P. 28049 Madrid, Spain; jmguisan@icp.csic.es; 5 Department of Chemical and Biological Engineering, Hoyt Laboratory, Princeton University, Princeton, NJ 08544, USA

**Keywords:** thermoalkaliphilic lipase, engineered lipase, engineered disulfide bond, interfacial activation, lipases activity enhancement

## Abstract

Enhancement, control, and tuning of hydrolytic activity and specificity of lipases are major goals for the industry. Thermoalkaliphilic lipases from the I.5 family, with their native advantages such as high thermostability and tolerance to alkaline pHs, are a target for biotechnological applications. Although several strategies have been applied to increase lipases activity, the enhancement through protein engineering without compromising other capabilities is still elusive. Lipases from the I.5 family suffer a unique and delicate double lid restructuration to transition from a closed and inactive state to their open and enzymatically active conformation. In order to increase the activity of the wild type *Geobacillus thermocatenulatus* lipase 2 (BTL2) we rationally designed, based on its tridimensional structure, a mutant (*cc*BTL2) capable of forming a disulfide bond to lock the open state. *cc*BTL2 was generated replacing A191 and F206 to cysteine residues while both wild type C64 and C295 were mutated to serine. A covalently immobilized *cc*BTL2 showed a 3.5-fold increment in esterase activity with 0.1% Triton X-100 (2336 IU mg^−1^) and up to 6.0-fold higher with 0.01% CTAB (778 IU mg^−1^), both in the presence of oxidizing sulfhydryl agents, when compared to BTL2. The remarkable and industrially desired features of BTL2 such as optimal alkaliphilic pH and high thermal stability were not affected. The designed disulfide bond also conferred reversibility to the enhancement, as the increment on activity observed for *cc*BTL2 was controlled by redox pretreatments. MD simulations suggested that the most stable conformation for *cc*BTL2 (with the disulfide bond formed) was, as we predicted, similar to the open and active conformation of this lipase.

## 1. Introduction

Lipases (triacylglycerol acyl hydrolases, E.C. 3.1.1.3) are one of the most widely used enzymes in the industry due to their stability, broad substrate specificity, high selectivity, and advantageous promiscuity in some cases [1,2,3]. The cleaning industry has one of the highest demands for lipases, as alkaline lipases are part of the enzymatic additives intended to increase detergent efficiency and sustainability. The improvement of lipase activity and stability in the presence of detergents is a major goal and recurrent topic in this industry [4,5]. Lipases often are in a thermodynamic equilibrium between closed and open conformations, the latter being the enzymatically active state, which is found in the presence of water–oil interfaces, substrate analogs, and micelles [6,7,8]. Transition from closed to open conformation requires the movement and sometimes the structural rearrangement of a helical lid that hinders the active site [6,7,8]. Several strategies have been developed to increase lipase enzymatic activity, including immobilization on hydrophobic supports, chemical modification, site-directed mutagenesis, and medium engineering (presence of additives as detergents or organic solvents) or the use of *Combi*-lipases for the transformation of natural triglycerides. In general, these approaches are designed to decrease steric hindrance in the access of the substrate into the enzyme active cleft [9,10,11,12,13]. The introduction of disulfide bonds represents another strategy to modulate the enzymatic properties of lipases. The insertion of cysteine residues at a distance to form a disulfide bond in the core of lipases from *Rhizomucor miehei* and *Yarrowia lipolytica*, or in the lid hinge region of the *Rhizopus chinensis* lipase increased their thermostability and for the latter, also altered the length specificity of the acyl chains that this enzyme is capable to bind and hydrolyze [14,15,16]. While there are bioinformatic tools to facilitate the design and application of such strategy [17], the rational design and implementation of disulfide bonds to control and increase the hydrolytic activity of lipases, without compromising other enzyme features, remains elusive.

The lipase 2 of *Geobacillus thermocatenulatus* (BTL2) is a thermoalkaliphilic enzyme (part of the I.5 family of lipases) whose interesting properties, such as high thermal stability, high substrate selectivity, and tolerance to alkaline pHs, have been explored for biotechnological application [18]. The structure of BTL2 defined the unique conformational changes that the I.5 family of lipases suffer upon activation, including a characteristic double lid that controls the transition between the open (active) and closed (inactive) conformations [7,18]. This double lid consists in a main lid (residues 169 to 200) that changes its secondary structure during the process of interfacial activation revealing the active site groove (open conformation), and a secondary lid (residues 217 to 234) that plays the role of other common lipase lids, and that in BTL2 structurally supports the main lid and helps stabilizing the enzymatically active state [5]. Several strategies have been applied to enhance its catalytic properties, including chemical modifications with small molecules, polymers, peptides, and/or through molecular biology tools [19,20,21,22,23]. Thus, its enzymatic activity is substantially impacted when the double lid of BTL2 is modified [13,19,23,24]. 

**Figure 1:**
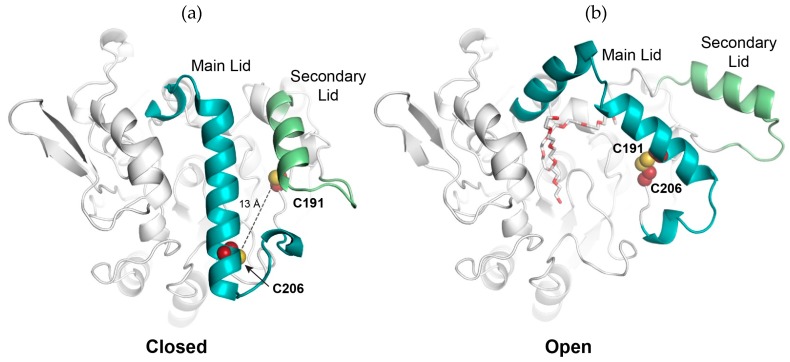
Design and modeled disulfide bond in co-crystallized wild type *Geobacillus thermocatenulatus* lipase 2 *(cc*BTL2). (**a**) Model of the closed conformation of *cc*BTL2 (using wild type *Geobacillus stearothermophilus* lipase 1 (BSL1) as template). The residues C206 and C191 (red and yellow spheres) are 13 Å away from each other (dotted line). The main and secondary lids are labeled (blue and green cartoons respectively). (**b**) Model of the open conformation of *cc*BTL2 based on the crystallographic structure of BTL2 in complex with Triton X-100 moieties (red and white sticks). In this conformation the cysteine residues are within an interaction distance to form a disulfide bond keeping ccBTL2 in an active conformation.

Based on the known open conformation of BTL2 [7] and a model of its closed state using the homologous (95% identity) L1 from *Geobacillus stearothermophilus* (also part of the I.5 family) as a template [25], we designed a quadruple mutant (A191C, F206C, C64S, and C295S) (called henceforth *cc*BTL2 in this study), containing only two cysteine residues (Figure S1). These cysteine residues are secluded and structurally far away in the closed conformation, but in proximity and able to interact and form a disulfide bond as they approach in the open state (Figure 1). This disulfide bond was designed to act as a physical restraint that would keep the lipase in its active conformation. The previously reported free cysteine BTL2 mutant (C64S, C295S) (based on the pT1BTL2 plasmid [26]) was used to produce *cc*BTL2 by including the mutations A191C and F206C [27]. Since soluble lipases are prone to form aggregates, CNBr-Sepharose^®^ conjugated derivatives of *cc*BTL2 and BTL2 were made to avoid artifacts resulting from intermolecular interaction during the characterizations [28,29,30]. The effect of the presence of surfactants and thiol-disulfide redox pretreatments, among others, on the activity of insoluble preparations of BTL2 and *cc*BTL2, were determined. The activation mechanism of the mutant and wild type enzyme was analyzed by targeted molecular dynamics (TMD) to structurally enlighten the observed behavior [31].

## 2. Results and Discussion

### 2.1. Design of An Engineered BTL2 Mutant (ccBTL2) to Lock the Open Conformation

According to the open structure of BTL2 co-crystallized with two molecules of Triton X-100 (PDB code: 2W22) and the closed conformation of a highly homologous *Geobacillus stearothermophilus* L1 (BSL1) (sharing 95% identity, PDB code: 1JI3), the residues F206 and A191 are reasonably far from each other (13 Å) and structurally secluded in the inactive (closed) state but nearby (3.5 Å) and partially exposed in the active (open) conformation (Figure 1). The residue F206, at the core of the protein, remains relatively immobile during the close-to-open transition and helps anchoring the conformation of the secondary lid in the closed state. On the other hand, residue A191, belonging to the α-helix that covers the active site pocket (and part of the main lid), suffers a considerable displacement (around 20 Å) between active and inactive conformations. Thus, we hypothesized that replacement of these residues by cysteine would, under certain conditions (e.g., presence of Triton X-100 and oxidizing agents), induce the formation of a disulfide bond (C191–C206) in the open conformation, that could stabilize and lock the active state of the enzyme.

To avoid undesirable disulfide bond formation between wild type cysteine residues, C64, and C295 were previously mutated to serines obtaining the pT1øBTL2 plasmid we used as template [27]. We modeled the closed conformation of the designed mutant *cc*BTL2 (Figure 1) using the automated homology modeling program (ESyPred3D web server 1.0, www.fundp.ac.be/sciences/biologie/urbm/bioinfo/esypred/) [32], and with the *Geobacillus stearothermophilus* L1 lipase BSL1 (from the same I.5 family) in the closed conformation as template (PDB code: 1KU0). Sharing 95% identity, their tridimensional structure was basically identical having only few substitutions. The *cc*BTL2 open conformation was obtained by manually modifying the BTL2 open structure (2W22) to include the F206C and A191C mutations, followed by an energy minimization. According to these models, and as expected, the restructuration of the lids brings together the residues C191–C206 that would facilitate the formation of the disulfide bond in an enzymatically active conformation (open). The structural alignment of BTL2 and *cc*BTL2 models showed that no major structural changes are introduced by the F206C and A191C mutations (RMSD = 0.101 Å).

### 2.2. ccBTL2 Conserved Similar Expression and Optimal Properties 

As previously reported for other BTL2 mutants [21,22], the *cc*BTL2 recombinant expression was similar to that of BTL2 enzyme (7763 versus 7502 IU of *p*-NPB esterase activity/g wet cells, respectively) using the same expression system [26,27]. A SDS-PAGE with samples of both purified *cc*BTL2 and BTL2 showed a single ~43 kDa band for each enzyme (Figure S2). The purified *cc*BTL2 mutant, without redox pretreatment, had a specific activity (630 IU/mg) similar to that of the BTL2 (660 IU/mg) (Table 1). 

Immobilization of the enzymes onto CNBr-Sepharose^®^ resin showed similar activity recovery for both BTL2 (65%) and *cc*BTL2 (67%). No significant differences were observed for the optimal activity in terms of thermal stability (at 75 °C) and pH (7.0). Table 1 resumes similar overall properties of both enzymes and their immobilized derivatives and shows that the commonly desirable properties of the thermoalkaliphilic I.5 family were not affected in our engineered *cc*BTL2.

### 2.3. A Tunable Formation of the Disulfide Bond in ccBTL2 

As *cc*BTL2 was thermodynamically and biochemically comparable to BTL2, we then characterized the effect of redox pretreatments to control the formation of the disulfide bond in *cc*BTL2 and its activity. The enzymes were immobilized CNBr-Sepharose^®^ resin to reduce artifacts from protein–protein interactions (e.g., through their free β-thiol groups). To follow the formation of the disulfide bond we used 4-PDS to monitor the oxidation state of the cysteine residues. This compound interacts with the reduced state of the S groups (-SH) releasing 4-pyridinethiol that after tautomerization absorbs at 324 nm [33]. The 4-PDS was added after no redox treatment, or after either a reducing treatment with DTT (or glutathione) or oxidizing treatment with Cu^2+^ (or oxidized glutathione). Increment in absorbance at 324 nm indicates the presence of –SH groups and therefore no disulfide bond formed. The untreated *cc*BTL2 derivative showed lower A_324nm_ values (~0) than those of BTL2, suggesting that β-thiol groups were already oxidized and the disulfide bond formed in the purified protein. During the extraction and purification of *cc*BTL2, the enzyme was exposed to both detergents and oxygen (exposed to air) that could trigger the adoption of the open conformation and oxidize the cysteine residues, respectively, favoring the formation of the disulfide bond when the residues were in close proximity. In addition, it has been reported that disulfide bonds can be obtained “spontaneously” when natural or synthetic peptides are dissolved in aqueous medium (distinctin, CXCR4 inhibitors) and exposed to air as long as they previously achieve the correct native conformation [34]. Disulfide bond formation can also be promoted by Zn(II), Fe(II), Cd(II) or Sulfur when present even as traces in the culture media, buffers or in agarose based materials as those used in our study [35]. Our results clearly indicate a formation of the disulfide bridge C206-C191 in the purified *cc*BTL2 previous to any redox treatment, which we believe was very likely promoted after extraction and purification. It is noteworthy that perhaps no pretreatment would be necessary for eventual industrial applications of such BTL2 mutant.

Next, we wanted to control and test the reversibility of the disulfide bond formation. We then used DTT as reducing agent and Cu^2+^ as catalyst for oxidation, which were more effective than glutathione and oxidized glutathione, respectively. In both cases denaturing conditions during the application of the treatment (using guanidine) increased the effectiveness of pretreatments (Table 2) probably by promoting higher exposition of β-thiol groups [36]. DTT (25 mM), triggered the highest values of A_324nm_ for both *cc*BTL2 and BTL2, suggesting a high degree of free β-thiol groups (>90%) in both immobilized enzymes according to the expected maximum A_324nm_ value (~0.74). When the reduced immobilized enzymes were then incubated under oxidizing conditions using Cu^2+^, A_324nm_ returned to values close to zero (<0.1), indicating that now the β-thiol groups were effectively re-oxidized showing a precise degree of control and reversibility of the system. A total of five oxidation-reduction cycles were repeated using Cu^2+^ and DTT (200 µM and 25 mM, respectively). BTL2 had an irreversible oxidation after three cycles with A_324_ values remaining close to zero. In contrast, A_324_ values for *cc*BTL2 were essentially the same after a fifth cycle for either the oxidation step (~0) or reduction step (~0.7) demonstrating a higher tolerance to the redox treatment.

### 2.4. Activity of the Engineered ccBTL2 Is Enhanced in Presence of Detergent

#### 2.4.1. Effect of Cu^2+^ Pretreatment in Enzymatic Activity in Absence of Detergents

**Figure 2:**
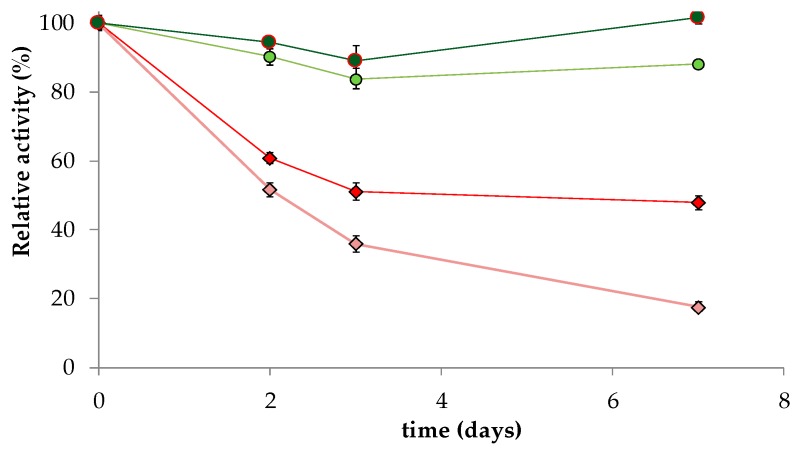
Effect of 20 µM Cu^2+^ (dark green and red) and 200 µM Cu^2+^ (light green and pink) incubations at pH 7.50 and 30 °C in presence of 0.1% (*w*/*v*) Triton X-100 on the relative hydrolytic activity of CNBr derivatives of *cc*BTL2 (circles) and BTL2 (rhombus). Initial derivative activity was 5 IU (*p*-NPB) and 0.013 mg of protein/g.

Immobilized *cc*BTL2 activity remains unaltered upon incubation with low concentrations of Cu^2+^ (20 µM), even when β-thiol groups are oxidized (Figure 2), and showed slower inactivation than BTL2 upon incubation with higher concentrations of Cu^2+^ (200 µM) (Figure 2). 

This is consistent with previous findings for free BTL2 and the homolog *Geobacillus stearothermophilus lipase* 1 (BSL1), both inactivated in the presence of Ag^+^ and Cu^2+^, which act as catalyzers in the oxidization of β-thiol groups [37,38]. Oxidation of wild type Cys residues can explain the DTT mediated reactivation of immobilized BTL2, which was inactivated after denaturation in guanidine [39]. *cc*BTL2 was stable and with similar activity after treatment with guanidine, an effect that is probably a consequence of the high stability provided by the disulfide bond being previously formed.

#### 2.4.2. Hydrolysis of *p*-NPB in Presence of Detergents

**Figure 3:**
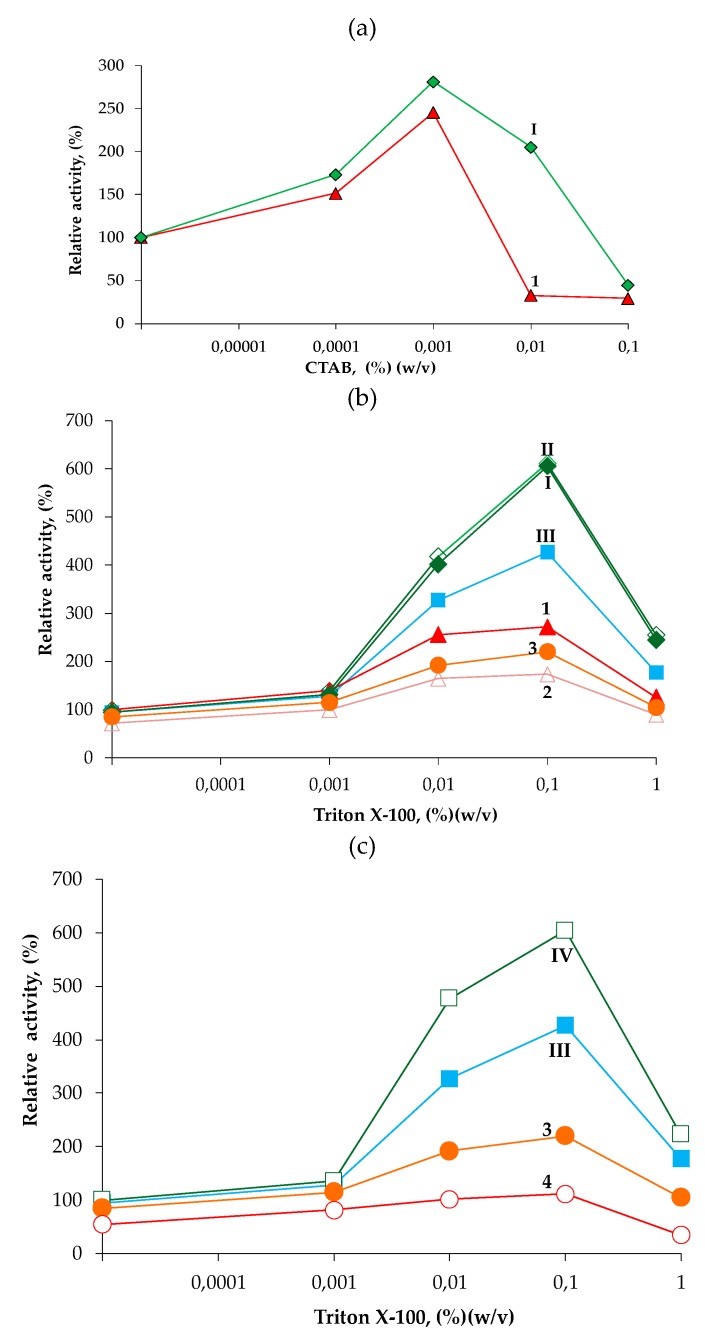
Effect of hexadecyltrimethylammonium bromide (CTAB) (**a**) on the hydrolytic activity of unmodified CNBr derivatives of BTL2 (“1” or red triangles) and *cc*BTL2 (“I” or green rhombus). Effect of sequential pretreatments on hydrolytic activity of CNBr lipase derivatives in the presence of Triton X-100. (**b**): (“I” or green rhombus) *cc*BTL2 and (“1” or red triangles) BTL2 as obtained; (“II” or open green rhombus) *cc*BTL2 and (“2” or open pink triangles) BTL2 after incubation with 200 µM Cu^2+^ (first treatment). (b) and (**c**): (“III” or solid cyan squares) *cc*BTL2 and (“3” or solid orange circles) BTL2 after posterior incubation with 25 mM DTT (second treatment). (c): (“IV” or open green squares) *cc*BTL2 and (“4” or open red circles) BTL2 re-incubated with 200 µM Cu^2+^ (third treatment). Values are relative to the respective unmodified derivative activity (5 IU *p*-nitrophenyl butyrate (*p*-NPB) and 0.013 mg of protein/g) in the absence of detergents for *cc*BTL2.

Lipases are key additives for the washing industry [4,5]. Among their applications, optimal concentrations of detergents can improve free or immobilized lipases catalytic activity, most probably by disaggregation of oligomeric forms, stabilization of the active enzyme conformation, and/or facilitating substrate accessibility and dissolution [40,41]. Thus, we assessed the effect of detergents on the hydrolytic *p*-NPB activity of immobilized BTL2 and *cc*BTL2. Both immobilized enzymes without detergent showed a similar *p*-NPB hydrolytic activity (Table 2). However, with 0.01% CTAB and as obtained after immobilization, *cc*BTL2 showed enhanced activity (~200%) (*w*/*v*) while the wild type immobilized enzyme showed an opposite effect, a reduction to 33%, meaning that ccBTL2 was 6-fold higher increment (Figure 3a; I versus 1). On the other hand, with the addition 0.1% (*w*/*v*) Triton X-100 (Figure 3b; I versus 1) the relative activity increment was again higher for ccBTL2 (605%) than for BTL2 (235%). Is important to note that these results were obtained without using any redox pretreatment, which again indicates a very likely oxidized state of the *cc*BTL2 as the 4-PDS assay indicated.

Reversibility of the system was tested by the application of sequential oxidation-reduction-oxidation cycles while measuring the activity of conjugated lipases at different concentrations of Triton X-100. In Figure 3b (II versus 2) it is shown that the initial oxidizing pretreatment (with 200 µM Cu^2+^) did not cause substantial effects on the activity of *cc*BTL2 (maximum was 611%) while for the BTL2 the oxidizing treatment causes a general activity decrease. The maximum activation value for BTL2 went from 272% to 174% at a concentration of 0.1% of Triton X-100, implying that ccBTL2 has 3.5-fold higher activity increment after this pretreatment. The subsequent pretreatment of *cc*BTL2 derivative at reducing conditions (25 mM DTT) caused a loss of relative activity (maximum value went from 605% to 426%) while for the wild type the reducing pretreatment caused a partial recovery from 174% to 221% (Figure 3b or 3c; III versus 3). The final oxidation pretreatment (Figure 3c; IV versus 4), brings back *cc*BTL2 to the initial high relative activity values (604%). At this point, BTL2 showed (in the re-oxidation step) evidence of cumulative inactivation (relative activity value was just 112%). These results were in agreement with the 4-PDS measurements after the same pretreatments (Table 2).

All the above confirmed that the designed disulfide bond C191-C206 was already established in the unmodified *cc*BTL2 after extraction and purification. Both unmodified and re-oxidized *cc*BTL2 showed the higher relative activity (up to 5.4 times) in the presence of detergents when compared to BTL2. The lack of this engineered disulfide bond may also explain the decreased activity caused by 0.01% CTAB (Figure 3b) or by Cu^2+^ oxidative incubation for the BTL2 (Figure 2) for which the oxidation of native cysteine residues has been related before with a less active enzyme form [39]. Our engineered *cc*BTL2 showed higher activity in the presence of detergents and also higher resistant to the oxidation-reduction cycles while none of the common negative side effects on the activity, in oxidizing conditions, known for the BTL2 [37,38]. This tendency was also seen with other *p*-NP esters of different acyl length chain (Figure S3).

#### 2.4.3. Transformation of Natural Triglycerides

Both immobilized (BTL2 and *cc*BTL2) enzymes were then compared, as obtained from purification or after oxidative or reducing treatments, for the hydrolysis of industrially relevant substrates like sardine oil and in the ethanolysis of palm-olein with ethanol (Table 3).

As seen with *p*-NP esters (Figure S2), the activity of both BTL2 immobilized enzymes against triglycerides was similar, either as obtained after purification or after reductive pretreatments with DTT. However, the oxidative pretreatment was highly detrimental for the BTL2 activity but not for *cc*BTL2. No significant changes in the EPA/DHA specificity were observed among derivatives regardless of the pretreatment. The low ethyl esters yield values seen in the ethanolysis of palm olein with ethanol were also seen for BTL2 immobilized on glyoxyl supports under mild reaction conditions [43], which in that specific reaction context discards its use as part of more complex catalytic systems as *Combi*-lipases [44].

### 2.5. Exploring ccBTL2 Active Conformation by Molecular Dynamics

To elucidate the possible conformation(s) adopted by *cc*BTL2 with the introduction of the mutations A191C and F206C and the formation of the disulfide bond, we generated simulations and models of both BTL2 and *cc*BTL2.

**Figure 4:**
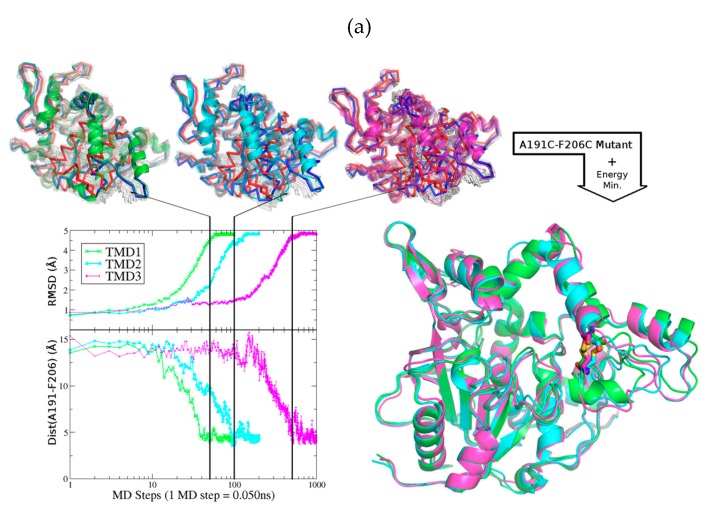

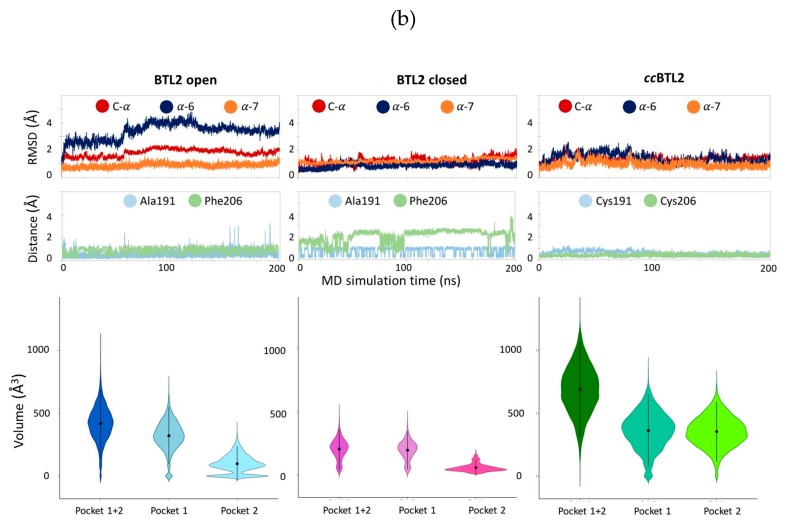
Analysis of the trajectories and conformations during the closed-to-open transition of *cc*BTL2. (**a**) (Bottom-left) Plot showing the root-mean-square deviations (RMSD) and distance between A191 and F206, of the structure generated along the targeted molecular dynamics (TMD) from the closed/inactive conformation towards the open/active conformation. (Top) Selected structures among the ensemble of intermediate conformations obtained from the TMD. (Bottom-right) In silico models of the *cc*BTL2 after energy minimization. (**b**) Evolution of the RMSD of the entire C-α backbone (red), α-6 helix (blue), α-7 helix (orange), and in light blue and green the RMSD of the entire amino acid Ala191 and Phe206 and their corresponding mutant during the 200 ns MD simulation. *Violin* plots of pocket exposure obtained from the Mdpocket software in Å^3^; the EGC-404 molecule from the PDB code 2W22 has taken as reference for Pocket 1, EGC403 for Pocket 2, and the summary of both for Pocket 1+2. Mean values and standard deviation are also plot (black dots and solid lines).

Since we lacked an initial structure in the closed conformation for BTL2, we generated a reliable homology model (model quality score: Of 0.66) [45]. The model was based on the crystallographic structure of the highly homologous L1 lipase from *Bacillus stearothermophilus* also belonging to the I.5 family and which, with a 95% sequence identity, gave us enough confidence on the similitudes of the structural conformations both enzymes adopt [46]. Next, we studied the conformational change between the open (active) and closed (inactive) conformations using targeted molecular dynamics (TMD) simulations [47], which determines the transition between conformations by applying standard molecular dynamics (MD) with a modified molecular mechanics force field. The approach employs steering forces dependent on the root mean square distance between the initial state and the target conformation scaled by a force constant. Three TMD simulations were generated from the closed/inactive model towards the open/active conformation found in the crystallographic structure of BTL2 (PDB code: 2W22). Different simulation times and force constants (5, 10 and 50 ns of simulation time with force constants of 1, 0.75 and 0.5, respectively) were used. We monitored the distance between the amino acids A191 and F206 and observed that, over the three trajectories generated, only when the structure was near the active form, the distance would be short enough (around 5 Å distance between the alpha carbons) to allow the formation of the engineered disulfide bond on *cc*BTL2 (Figure 4a-Bottom-left). At this point, we took three different states from the TMD trajectories when the distance was at the required length for the disulfide bond formation and performed the mutations (Figure 4a-top) defining the disulfide bond in silico. Energy minimizations lead to three similar structures that maintained the secondary structure observed in the wild type form (Figure 4a-Bottom-right).

Having a probable conformation for the *cc*BTL2 mutant form, we then aimed to explore the stability and structural features of this model compared to the wild type open and closed conformations. 200 ns MD simulations were performed for each of the three molecular structures monitoring the RMSD of the alpha carbons atoms of the whole protein, and also the ones belonging to lids, the main responsible for the conformational changes (Figure 4a) [7]. As expected, we observed larger fluctuations on the open form compared with the closed conformation since the simulation was performed in an aqueous environment and the open conformation tends to fluctuate avoiding the lipophilic environment of the pocket. Nevertheless, *cc*BTL2 was stable for both overall RMSD and particularly the lids, even with lower fluctuations than the BTL2 in open conformation. We also checked any modifications of the volume of the active site groove (Figure 4b), defined by the region where the two lipid moieties are found in the crystallographic structure of BTL2 (Figure S4) [7]. Surprisingly, we found that the pocket of the *cc*BTL2 mutant rather than maintaining the exposed volume of the BTL2 in an open conformation, it increased with an average value of nearly 700 Å^3^ compared to the 450 Å^3^ observed in BTL2, reaching maximum values of nearly 1500 Å^3^. Both, the intermediate state similar to the active conformation reached by *cc*BTL2 and its apparent higher stability could explain the increment in activity while in the presence of detergents.

## 3. Materials and Methods

### 3.1. Materials

The BCA Protein Assay Kit was from Pierce^®^ (Rockford, IL, USA). Cyanogen bromide activated Sepharose^®^ 4BCL (CNBr) and butyl Sepharose^®^ CL-4B were purchased from General Electric (Upsala, Sweden). Ethanol, Dimethyl sulfoxide (DMOS), Ethanolamine hydrochloride, hydroxylamine hydrochloride, hexadecyltrimethylammonium bromide (CTAB), Triton X-100, Guanidine hydrochloride, dithiothreitol (DTT), 4,4′-dipyridyldisulfide (4-PDS), oxidized (GSSG) and reduced glutathione (GSH). 1,4-dioxane, *p*-nitrophenyl butyrate (*p*-NPB) and other *p*-NP esters (acetate, octanoate, dodecanoate and hexadecanoate), CuCl_2_·5H_2_O, ethylenediaminetetraacetic acid (EDTA) and salts for buffering solutions were purchased from Sigma-Aldrich (St. Louis, MO, USA). Palm olein was bought in a local store. The sardine oil was a gift from BTSA, Biotecnologías Aplicadas, S.L. (Madrid, Spain). 

### 3.2. Site Directed Mutagenesis, Cloning, Expression and Purification of BTL2 Variants

Site-directed mutagenesis, cloning and experiments were made according to our previous work [27,48,49]. The previously constructed øBTL2 mutant pT1C64S, C295SBTL2 plasmid (lacking wild type cysteine codons) [27] was used as template to construct the pT1A191C øBTL2 plasmid by site-directed mutagenesis using the following primers: Forward GCGGTGTTGAAAGCGtgcGCTGTCGCCAGCAAT and reverse ATTGCTGGCGACAGCgcaCGCTTTCAACACCGC. This pT1A191C øBTL2 plasmid was used then as template to construct the final pT1A191C-F206C øBTL2 plasmid using primers: Forward AGTCAAGTATACGATtgcAAGCTCGACCAATGG and reverse CCATTGGTCGAGCTTgcaATCGTATACTTGACT. Mutations were verified by sequencing. Transformation of *E. coli* BL21(DE3) cells, expression, and purification of BTL2 variants were performed as stated before [27,48,49]; all the plasmids mentioned here are based on and derived from the initial pT1-BTL2 vector [26].

### 3.3. Protein Determination, Enzymatic Activity Assay, Enzyme Immobilization and Characterization of Derivatives

Protein determination (BCA pierce method of high sensitivity) and esterase activity against *p*-NPB of soluble or immobilized BTL2 variants at different pHs and temperature were determined as described before [48]. The *p*-NPB assay in presence of different concentrations of Triton X-100 or CTAB was carried out as previously described [19]. An international unit (IU) of *p*-NPB hydrolysis is defined as the amount of enzyme necessary to hydrolyze one µmol of *p*-NPB/min at pH 7.0 and 25°C. The preparation of CNBr enzyme derivatives (5 IU/g or 6600 IU/g with a protein content of 0.013 and 10.7 mg, respectively), and determination of optimal pH, temperature and stability assays were carried out as reported before [18].

The effect of the acyl chain length of *p*-NP esters on the enzyme activity was determined using a method reported previously [23] with minor modifications: The final substrate concentration was 0.4 mM in 20% DMSO (80% aqueous) in 25 mM tris-HCl buffer pH 7.0 plus 0.01% (*w*/*v*) Triton X-100 at 25°C. The IU esterase activity is defined as the amount of enzyme necessary to hydrolyze one µmol of *p*-NP ester/min under the described conditions.

### 3.4. Assay with 4-PDS for CNBr Derivatives

The assay was performed as described before [27,50] with modifications: A sample of CNBr enzyme derivatives (6600 IU/g) was added to obtain the equivalent of 5 mg of protein (according to the BCA method) and suspended in a solution containing 1 mM 4-PDS, 1 mM EDTA, and 8 M guanidine hydrochloride in a 50 mM sodium phosphate buffer at pH 7.5 (final volume of 5 mL) [39]. After 1 h, the liberation of 4-thiopyridone as a result of thiol-disulfide exchange was measured at 324 nm against the supernatant control with the same composition but using blocked CNBr-Sepharose^®^ (treatment with 2 M ethanolamine hydrochloride at pH 8.0) instead of the lipase derivatives. The calibration curve was made with L-cysteine (From 10 µM to 100 µM) in the control medium.

### 3.5. Incubation of CNBr Derivatives in Oxidizing/Reducing Conditions

#### 3.5.1. Oxidizing Conditions

Conditions to promote disulfide bond formation [36,38] were set when 5 g of CNBr derivatives of lipases (made with 5 IU/g or 6600 IU/g) were suspended at 30°C in 50 mM sodium phosphate buffer at pH 7.5 containing glutathione redox pair (10 mM oxidized/1 mM reduced) or Cu^2+^ at 20 μM (or 200 μM concentration), and 0.1% Triton X-100 (*w*/*v*) in the presence or absence of guanidine hydrochloride 8 M to a final volume of 50 mL [39]. Experiments were carried out from one day to one week when needed. Next, CNBr derivatives were filtered and washed thoughtfully with 1 mM EDTA/25 mM sodium phosphate buffer at pH 7.0, then with 25 mM sodium phosphate buffer at pH 7.0 and used for enzymatic assays [39].

#### 3.5.2. Reducing Conditions

Five grams of CNBr derivatives of the enzymes (whether treated or not under oxidizing conditions) (5 IU/g or 6600 IU/g) were suspended at 30°C in 50 mM sodium phosphate buffer at pH 7.5 containing glutathione redox pair (1 mM oxidized/ 10 mM reduced) or 25 mM DTT and 0.1% Triton X-100 (*w*/*v*) in the presence or absence of guanidine hydrochloride 8 M to a final volume of 50 mL. Experiments were carried out from one day to one week when needed. After that, derivatives were washed as described above and immediately used for the enzymatic assay [39].

### 3.6. Derivative Thermal Stability

The CNBr BTL2 derivatives were incubated over time in 25 mM sodium phosphate at 75°C and pH 7.0. Samples were withdrawn periodically using a pipet with a cut-tip and under vigorous stirring to have a homogeneous biocatalyst suspension [48]. The activity was measured using the *p*-NPB assay described above.

### 3.7. Transformation of Natural Triglycerides

The sardine oil hydrolysis and the ethanolysis of palm olein with ethanol catalyzed by BTL2 and *cc*BTL2 CNBr-Sepharose^®^ derivatives with 6600 IU/g was carried out as described previously [43,44] with the following changes: ethanol 96% and temperature at 25°C. All the experiments were carried out by triplicate and the standard error was under 5% unless stated otherwise.

### 3.8. Targeted Molecular Dynamics

Targeted molecular dynamics (TMD) was applied to understand the conformational changes of the transition of BTL2 from the closed to the open conformation. The information generated by TMD was then used in the analysis and calculation of the most likely conformation adopted by *cc*BTL2 once the cysteine mutations were included and the disulfide bond formed. The closed conformation of BTL2 was generated by homology modeling using as template the thermostable bacterial lipase L1 from *Bacillus stearothermophilus* [46] (PDB code: 1JI3), which, like BTL2, also belongs to the I.5 family of lipases. The open conformation of BTL2 was previously solved in complex with Triton X-100 molecules within its active site [7] (PDB code: 2W22). Three trajectories were generated using different parameters (Supplementary material, Section 1). Distances between A191 and F206 were monitored leading to intermediate conformations where the formation of disulfide bond was possible after including the cysteine mutations in both residues to create *cc*BTL2. The resulting structures of *cc*BTL2 with the formed disulfide bond were similar but not identical to the open conformation observed in the crystallographic structure of BTL2 (Figure 4a). Classical MD simulations were also performed in BTL2 (closed and open conformation) and *cc*BTL2 during 200 ns for further RMSD studies and pocket stability (Supplementary Material, Section 1).

## 4. Conclusions

In order to increase the activity of the *Geobacillus thermocatenulatus* lipase 2 (BTL2) we hypothesized that the rational insertion of two cysteine residues in specific positions (of the main lid and core of the protein) would allow us to conditionally control the level of aperture of the enzyme. The aperture is naturally controlled by a double lid restructuration to transition from a closed (inactive) state to their open and enzymatically active conformation. This conditional control, which depends on the redox conditions, would facilitate the entrance and hydrolysis of the substrate by keeping the enzyme in an active conformation. By means of a structurally-based rational design, we created the quadruple mutant *cc*BTL2 (A191C, F206C, C64S, and C295S), which proved effective to increase the esterase activity by 3.5-fold in the presence of 0.1% Triton X-100 (2336 IU mg^−1^) and up to 6.0-fold increment with 0.01% CTAB (778 IU mg^−1^) both in the presence of oxidizing sulfhydryl agents when compared to the wild type enzyme BTL2. The insertion of this disulfide bond did not affect the specific activity, and most importantly, neither the stability of the enzyme nor the delicate transition between conformations (open to closed). In fact, *cc*BTL2 resulted in a redox-tunable enzyme that supported several oxidation-reduction cycles that the wild type BTL2 (even in the presence of detergents) did not. All of our experimental data unequivocally indicated that the oxidized form of *cc*BTL2, which carries the engineered disulfide bond C191-C206, was already established during extraction and/or purification of the enzyme. TMD simulations suggested a stable conformation for *cc*BTL2 once the engineered disulfide bond was formed. As we predicted, this conformation was structurally similar to the crystallographic open conformation previously observed, but having an active site with bigger volumetric capacity for both of the grooves that stabilize the acyl chains of the substrates. To our knowledge, this is the first report of a lipase engineered with a disulfide bond in the lid that effectively increases its activity [15], without compromising any other of the industrially relevant features including thermostability or tolerance to alkaline pHs that are shared by the I.5 family of lipases to which BTL2 does belong.

## Figures and Tables

**Table 1:** Properties of BTL2 and *cc*BTL2 CNBr-Sepharose^®^ derivatives with 5 IU and 0.013 mg of protein/g.

Enzyme	Specific *p*-NPB Esterase Activity (IU/mg) ^a^	Activity Recovery (%) ^b^	Optimal pH ^c^	Optimal Temperature (°C) ^d^	t_1/2_ at 75 °C (min) ^d^
BTL2	660 ± 21	65± 2.3	8.5-9.5	64 ± 3.1	28 ± 1.3
*cc*BTL2	630 ± 17	67 ± 3.1	8.5-9.5	66 ± 2.3	30 ± 1.1

^a^ At pH 7.00 in 25 mM sodium phosphate buffer at 25 °C with 0.40 mM of *p*-nitrophenyl butyrate (*p*-NPB) for soluble enzymes. ^b^ In regard to the correspondent control suspension of free enzyme and blocked CNBr-support (Cyanogen bromide-Sepharose^®^) [20]. ^c^ With 0.40 mM of *p*-NPB at 25 °C in sodium borate buffer. ^d^ In presence of 0.40 mM of *p*-NPB at pH 7.00 in 25 mM sodium phosphate buffer.

**Table 2:** 4,4′-dipyridyldisulfide (4-PDS) assay for CNBr-Sepharose^®^ derivatives (6600 IU of p-nitrophenylbutyrate (p-NPB)/g) after 24 h of different pretreatments.

Pretreatment in Presence (+) or Absence (-) of Guanidine 8 M			nmol of Equivalent Free Cys Per mg of Protein ^a^	
			BTL2	*cc*BTL2
*As obtained*		−	35.0 ± 2.26	0.752 ± 0.62
		+	7.01 ± 1.25	2.01 ± 0.02
*Reducing incubation*	25 mM DTT	−	34.8 ± 4.45	42.0 ± 1.75
		+	42.9 ± 1.88	44.0 ±2.26
	GSSG 1 mM/	−	31.4 ±2.63	32.4± 1.31
	GSH 10 mM	+	34.5 ± 3.13	38.4 ± 2.57
*Oxidizing incubation*	200 µM Cu^2+^	−	4.82 ± 1.38	6.08 ± 1.32
		+	4.51 ± 1.88	1.00 ± 0.81
	GSSG 10 mM/	−	19.6 ± 1.13	12.4 ± 4.40
	GSH 1 mM	+	2.63 ± 0.69	7.02 ± 2.00

^a^ Absorbance of the supernatant after suspending the correspondent pretreated derivative (with 4-PDS 1 mM at pH 7.50 under denaturing conditions (8 M guanidine) at a final volume of 5 mL. Blank was the supernatant resulting from the same experiment but using blocked CNBr-Sepharose^®^. GSSG (oxidized glutathione); GSH (reduced glutathione); dithiothreitol (DTT).

**Table 3:** Properties of immobilized BTL2 and ccBTL2 in the transformation of natural glycerides.

CNBr-Derivative	Treatment	Hydrolysis of Sardine Oil		Palm Olein Ethyl Esters Yield at 72 h (%) ^c^
		Activity ^a^(µmol PUFAs/min)	Specificity ^b^ (EPA/DHA)	
BTL2	As obtained	0.86 ± 0.04	1.9 ± 0.1	22.1 ± 1.9
	200 µM Cu^2+^	0.15 ± 0.02	2.0 ± 0.3	<5
	25 mM DTT	0.95 ± 0.06	1.8 ± 0.2	30.3 ± 2.9
*cc*BTL2	As obtained	0.96 ± 0.04	1.6 ± 0.2	29.5 ± 1.1
	200 µM Cu^2+^	0.99 ± 0.06	1.7 ± 0.1	34.4 ± 1.3
	25 mM DTT	0.85 ± 0.07	1.8 ± 0.2	25.7 ± 2.2

^a^ Reaction conditions and analysis were the same as described for the biphasic hydrolysis system [42] (cyclohexane/aqueous buffer at pH 7.0) at 25 °C using highly loaded derivatives (6600 IU *p*-NPB and with 10.7 mg of protein/g); the reactions were followed using reverse phase (RP)-HPLC with an ultraviolet (UV) (215 nm). ^b^ Specificity is defined as the molar eicosapentaenoic acid/docosahexaenoic acid EPA/DHA production rate ratio [42].^c^ Palm olein ethanolysis conditions was performed using the one-step solvent-free method (ethanol: oil molar ratio 3:1; catalyst at 6% of the total oil mass) but changing the temperature to 25 °C and using 96% ethanol, reaction was followed using FTIR-ATR (attenuated total reflection) [43,44].

## Supplementary Materials

Supplementary materials can be found at https://www.mdpi.com/1422-0067/20/21/5245/s1. 1. Molecular dynamic methods; Figure S1: Residues in BTL2 that were modified to obtain the quadruple mutant *cc*BTL2. Figure S2: SDS-PAGE electrophoresis of BTL2 and *cc*BTL2 lipases; Figure S3: Hydrolysis of *p*-NP esters of different acyl chain length with immobilized BTL2 and *cc*BTL2 with different redox pretreatments; Figure S4: Reference volumes used to monitor the variation between the crystallographic structures of BTL2 and the *cc*BTL2 model.

## Abbreviations

*BTL2*	Wild type *Geobacillus thermocatenulatus* lipase 2
øBTL2	BTL2 mutant lacking native cysteine residues (C64S, C295S BTL2)
*cc*BTL2	A191C, F206C, C64S, C295S BTL2 (engineered mutant)
CNBr	Cyanogen bromide Sepharose^®^ support
CTAB	hexadecyltrimethylammonium bromide
4-PDS	4,4′-dipyridyldisulfide
p-NPB	p-nitrophenyl butyrate
p-NP	p-nitrophenyl ester
DTT	Dithiothreitol
GSH	Glutathione (reduced form)
GSSG	Glutathione (oxidized form)
PUFAs	Polyunsaturated fatty acids
EPA	Eicosapentaenoic acid
DHA	Docosahexaenoic acid
TMD	Targeted Molecular Dynamics
