# Supplementary material for: Disulfide Engineered Lipase to Enhance the Catalytic Activity: A Structure-Based Approach on BTL2
Source: Int J Mol Sci. 2019 Oct 23;20(21):5245. doi: 10.3390/ijms20215245 (PMC6862113; doi:10.3390/ijms20215245)
Supplement: Supplementary file 1 [file ijms-20-05245-s001.pdf]

International Journal of *Molecular Sciences*

Supplementary material

INDEX

1. Molecular dynamic methods…………………………………………………………..…..Page 2.

2. Residues in BTL2 that were modified to create the quadruple mutant *cc*BTL2……Page 4.

3. Figure S2. SDS-PAGE electrophoresis of BTL2 and *cc*BTL2 lipases…………..……..Page 5.

4. Figure S3. Effect of the acyl length on the hydrolysis of *p*-nitrophenyl esters with immobilized BTL2 and *cc*BTL2 applying different redox pretreatments………………………………….Page 6.

5. Figure S4. Reference volumes used to monitor the variation between the crystallographic structures of BTL2 and the ccBTL2 model ……………………………...….Page 7.

6. References……………………………………………………………………………..Page 8.

1. Molecular dynamics methods.

The crystallographic structure of the BTL2 in open conformation (UniProtKB - Q59260) was obtained from the RCSB-Protein Data Bank (PDB code: 2W22). In order to prepare the structures for further simulations, water molecules and co-solvent molecules including both Triton X-100 molecules (EGC-403 and ECG-404) (Fig. S4) were removed. Next, we built a homology model of the BTL2 in the closed conformation using as template the *Bacillus stearothermophilus* lipase 1 (UniProtKB - Q9L6D3, PDB code: 1JI3) with a sequence homology of 95.1%. This homology model was built with the SWISS-MODEL web server [1] obtaining a positive QMEAN value [2] of 0.66. The protonation states were calculated for both structures using the H++ web server [3] which relies on AMBER parameters and finite difference solutions to the Poisson–Boltzmann equation.

The conformational change from the closed to the open conformation was simulated using targeted molecular dynamics (TMD) as implemented in AMBER 16 [4], using the previously generated models. A salt concentration of 0.15 M and an internal and external dielectric constant of 4 and 80, respectively, were used. Atom types and charges were assigned according to AMBER ff15FB (Force Balance) force field [5]. Both systems were hydrated by using boxes containing explicit TIP3PFB water molecules [6] with added counter ions to maintain electro neutrality. Solvent molecules and counter ions were relaxed by energy minimization and then allowed to redistribute around the positional restrained structures during a 50 ps run at constant temperature (300 K) and pressure (1 atm). These initial harmonic restraints were gradually reduced in a series of progressive energy minimizations steps until they were completely removed. The resulting systems were heated again from 100 to 300 K during 20 ps and allowed to equilibrate in the absence of any restraints for 1.0 ns during which the system coordinates were collected every 2 ps for further analysis. The equilibrated structures were then used as the starting points for the three targeted MD simulations we ran. These simulations had a duration of 5, 10 and 50 ns applying a steering force based on a mass-weighted RMSD with respect to reference target conformation with force constants of 1, 0.75 and 0.5. Periodic boundary conditions and the Particle Mesh Ewald methods were used to treat long-range electrostatic effects. The SHAKE algorithm [7] was used throughout; applied to all bonds and an integration step of 2.0 fs.

The Root mean square deviation of the c-alpha atoms (RMSD) was calculated and distances between A191 and F206 where measured along the TMD simulation with the help of ccptraj tool from Ambertools 14 [8]. Moreover, from each of the three trajectories, we selected a structure when residues A191 and F206 where at least at 5Å from each other; these structures were conformationally similar to the open conformation. Then, we built three *cc*BTL2 structures using the mutagenesis tool from PyMOL Molecular Graphics [9] and performed an energy minimization, which leaded to three highly similar models.

Finally, we performed molecular dynamic (MD) simulations on the *cc*BTL2, BTL2 open and closed conformations, in the absence of ligands using the previously generated molecular systems and AMBER16. The AMBER force field ff15FB (Force Balance) for the protein parametrization was applied. Water and metal parameters were obtained from the TIP3PFB and ions parameter modification file. After that, systems were minimized in vacuum, in order to release possible undesired interactions or clashes. Then the systems were embedded in an TIP3FB water box of approximately 12000 water molecules forcing neutrality by adding chloride ions. Initially, the

embedded systems were minimized and heated to 300 K in a NVT ensemble followed by equilibration during 0.5 ns in a NPT ensemble. In all systems the hydrogen atoms were kept at their equilibrium distance by means of the SHAKE algorithm. Atom pair distance cutoffs were applied at 10.0 Angstroms to compute the Van der Waals interactions, while long-range electrostatics were computed by means of Particle-Mesh Ewald (PME) method [10]. Finally, the MD simulation was performed up to 200 ns using the thermostat NPT ensemble at 300 K and generating snapshots each 20 ps for further analysis of both systems (10 000 in total). The trajectories of all complexes were collected and analyzed by the cpptraj module of AMBER16 [8] in order to obtain the previously mentioned root mean square deviation of the overall c-alpha atoms, and the atoms from alpha6- and alpha7-helices. Moreover, 10000 aligned PDBs were obtained from each trajectory to perform the analysis of the active pocket cavity by the versatile Fpocket software [11], which is based on Voronoi tessellation algorithm. The Mdpocket module [12] was applied to obtain the corresponding volumes in each MD simulation step using as a pocket reference (pocket1 and pocket2) the first ones obtained from the first step (md0) in the initial BTL2 in the open conformation. The volume of the pockets recorded along the simulation where represented as trend lines and *violin* plots using the software RStudio (www.rstudio.org) and the package ggplot2.

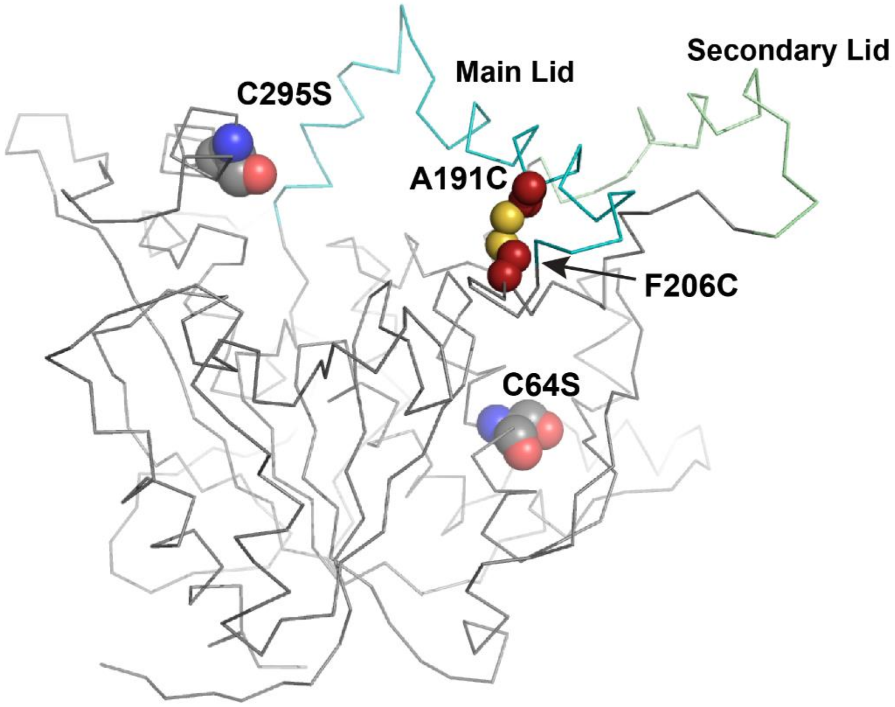

Figure S1. Residues in BTL2 that were modified to create the quadruple mutant *cc*BTL2.

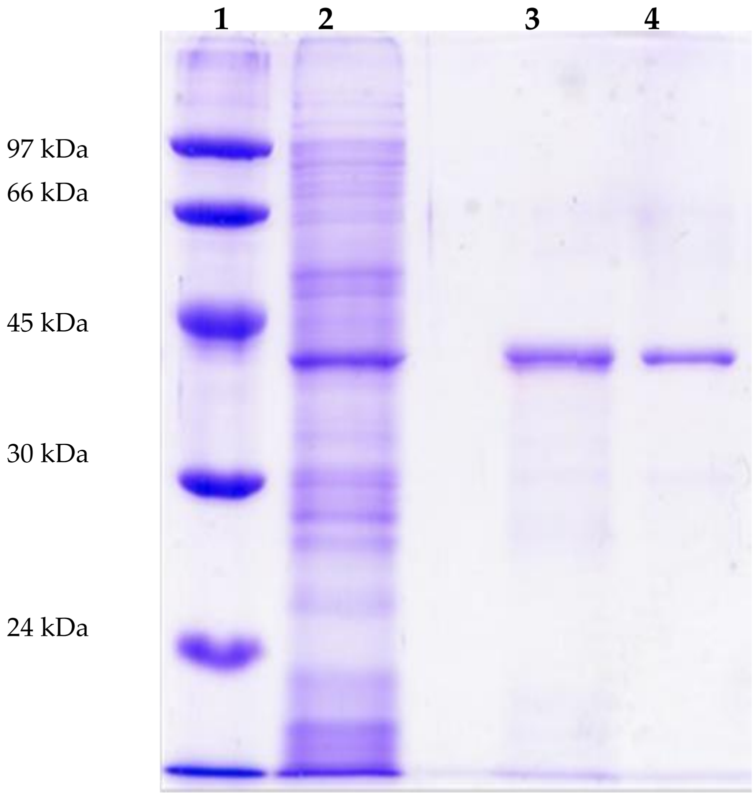

Figure S2. SDS-PAGE electrophoresis of BTL2 and *cc*BTL2 lipases. From left to right: Lane 1 (Molecular weight markers); Lane 2 crude extract from *E. coli* expressing ccBTL2; Lane 3 purified *cc*BTL2; Lane 4 purified BTL2.

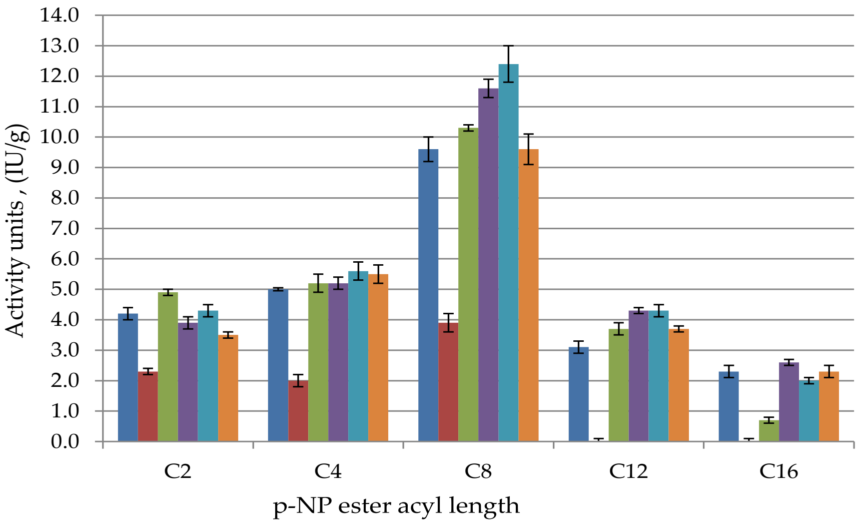

Figure S3. Hydrolysis of *p*-NP esters of different acyl chain length with immobilized *wt*BTL2 and *cc*BTL2 with different redox pretreatments. BTL2-CNBr: as obtained from purification (blue bar), pretreated with 200 mM Cu^{2+} (red bar), pretreated with 25 mM DTT (green bar). *cc*BTL2-CNBr: as obtained from purification (purple bar), pretreated with 200 mM Cu^{2+} (cyan bar), pretreated with 25 mM DTT (orange bar).

Note S1: For both CNBr derivatives made with lipase without any pretreatment, there are not significant differences on hydrolytic activity (Figure 4) except for the C8 ester where ccBTL2-CNBr is 18,8 % (11.4 IU/g) higher than that of the BTL2 derivative (9,6 IU/g). The profile obtained for the BTL2 derivative activity vs. *p*-NP ester length chain is close to that obtained for the free BTL2 enzyme. It is noteworthy that both BTL2 and *cc*BTL2 showed broad substrate specificity towards *p*-NP acyl esters of different length, processing chains from C2 up to C16. Nevertheless, it was also obvious the relative higher activity against the C8 ester (Ref. [22] in the main text). The oxidative pretreatment deteriorates BLT2 derivative activity especially with C12 and C16 p-NP esters while for the *cc*BTL2 the same pretreatment was slightly positive or neutral. In general, the hydrolytic behavior of the *cc*BTL2 derivative surpasses that of BTL2 even after the harsh oxidizing conditions assayed in presence of Cu^{2+}.

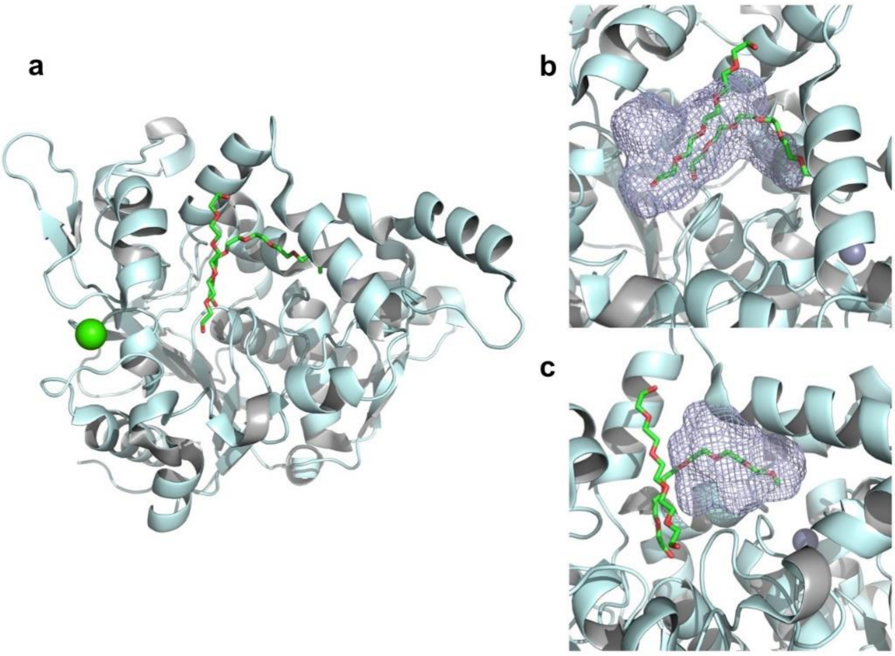

Figure S4. Reference volumes used to monitor the variation between the crystallographic structures of BTL2 and the ccBTL2 model. a) Crystallographic structure of BTL2 (open conformation) in complex with Triton X-100 (EGC-403 and EGC-404) moieties (PDB code: 2W22). b) Volumetric representation (gray mesh) of Pocket 1 fitting the EGC-404 molecule. c) Volumetric representation (gray mesh) of Pocket 2 fitting the EGC-403 molecule.

References

1. Waterhouse, A.; Bertoni, M.; Bienert, S.; Studer, G.; Tauriello, G.; Gumienny, R.; Heer, F.T.; de Beer, T.A.P.; Rempfer, C.; Bordoli, L.; et al. SWISS-MODEL: homology modelling of protein structures and complexes. *Nucleic Acids Research* **2018**, *46*, W296–W303.
2. Benkert, P.; Biasini, M.; Schwede, T. Toward the estimation of the absolute quality of individual protein structure models. *Bioinformatics* **2011**, *27*, 343–350.
3. Gordon, J.C.; Myers, J.B.; Folta, T.; Shoja, V.; Heath, L.S.; Onufriev, A. H++: a server for estimating pKas and adding missing hydrogens to macromolecules. *Nucleic Acids Research* **2005**, *33*, W368–W371.
4. D.A. Case, R.M. Betz, D.S. Cerutti, T.E. Cheatham, III, T.A. Darden, R.E. Duke, T.J. Giese, H.G.; A.W. Goetz, N. Homeyer, S. Izadi, P. Janowski, J. Kaus, A. Kovalenko, T.S. Lee, S. LeGrand, P. Li, C.; Lin, T. Luchko, R. Luo, B. Madej, D. Mermelstein, K.M. Merz, G. Monard, H. Nguyen, H.T. Nguyen, I.; Omelyan, A. Onufriev, D.R. Roe, A. Roitberg, C. Sagui, C.L. Simmerling, W.M. Botello-Smith, J.S.; R.C. Walker, J. Wang, R.M. Wolf, X. Wu, L.X. and P.A.K. (2016) AMBER 2016. University of California, San Francisco.
5. Wang, L.-P.; McKiernan, K.A.; Gomes, J.; Beauchamp, K.A.; Head-Gordon, T.; Rice, J.E.; Swope, W.C.; Martínez, T.J.; Pande, V.S. Building a More Predictive Protein Force Field: A Systematic and Reproducible Route to AMBER-FB15. *The Journal of Physical Chemistry B* **2017**, *121*, 4023–4039.
6. Wang, L.-P.; Martinez, T.J.; Pande, V.S. Building Force Fields: An Automatic, Systematic, and Reproducible Approach. *The Journal of Physical Chemistry Letters* **2014**, *5*, 1885–1891.
7. Ryckaert, J.P.; Ciccotti, G.; Berendsen, H.J.C. Numerical integration of the cartesian equations of motion of a system with constraints: molecular dynamics of n-alkanes. *Journal of Computational Physics* **1977**, *23*, 327–341.
8. Roe, D.R.; Cheatham, T.E. PTRAJ and CPPTRAJ: Software for processing and analysis of molecular dynamics trajectory data. *Journal of Chemical Theory and Computation* **2013**, *9*, 3084–3095.
9. L DeLano, W. Pymol: An open-source molecular graphics tool. *{CCP4} Newsletter On Protein Crystallography* **2002**, *40*.
10. Darden, T.; York, D.; Pedersen, L. Particle mesh Ewald: An N·log(N) method for Ewald sums in large systems. *The Journal of Chemical Physics* **1993**, *98*, 10089–10092.
11. Le Guilloux, V.; Schmidtke, P.; Tuffery, P. Fpocket: An open source platform for ligand pocket detection. *BMC Bioinformatics* **2009**, *10*, 168.
12. Schmidtke, P.; Bidon-Chanal, A.; Luque, F.J.; Barril, X. MDpocket: open-source cavity detection and characterization on molecular dynamics trajectories. *Bioinformatics* **2011**, *27*, 3276–3285.

© 2019 by the authors. Submitted for possible open access publication under the terms and conditions of the Creative Commons Attribution (CC BY) license (http://creativecommons.org/licenses/by/4.0/).
